# Misleading Mercedes-Benz sign: A case report of misdiagnosed cholecysto-hydatid cyst fistula in acute cholecystitis

**DOI:** 10.1016/j.ijscr.2024.110416

**Published:** 2024-10-04

**Authors:** Mohamed Ali Chaouch, Ahmed Hadj Taieb, Besma Gafsi, Mohamed Zayati, Sofien Gaied, Faouzi Noomen

**Affiliations:** a Department of Visceral and Digestive Surgery, Monastir University Hospital, Monastir, Tunisia; b Department of Intensive Care, Monastir University Hospital, Monastir, Tunisia; c Department of Radiology, Monastir University Hospital, Monastir, Tunisia

**Keywords:** Liver hydatid cyst, Cholecysto-hydatid fistula, Hydatid disease, Emergency surgery, Case report

## Abstract

**Introduction:**

Liver hydatid cysts represent a significant health concern globally, particularly in endemic regions like Tunisia. While they often lead to complications such as biliary fistulas, diagnostic errors can arise from radiologic signs like the “Mercedes Benz sign,” which indicates gas within the gallbladder. This report highlights the challenge of diagnosing a rare cholecysto-hydatid cyst fistula, where the presence of gas in the gallstones initially suggested a fistula.

**Case presentation:**

A 30-year-old female presented with right hypochondrium pain and fever. Ultrasound suggested cholecystitis and identified two cystic formations in liver segments IVb and VII. CT scan revealed intravesicular air bubbles, suggesting a cholecysto-hydatid fistula. Emergency surgery was performed. Intraoperatively, there was an acute cholecystitis. The liver hydatid cyst of segment IVb communicated with the biliary tree and there was no cholecysto-hydatid fistula. We performed a cholecystectomy, cholangiography, and a total pericystectomy for the two liver hydatid cysts. The postoperative follow-up was uneventful.

**Discussion:**

The “Mercedes Benz sign,” often indicating gas within gallstones, is rare but can mislead the diagnosis toward a cholecysto-hydatid cyst fistula. This case highlights the diagnostic challenge posed by this radiological feature, which led to initial suspicion of a fistula. Hydatid cysts, though common in endemic regions, can lead to diagnostic dilemmas, especially when atypical signs are present.

**Conclusions:**

The presence of gas in the gallbladder can mislead the diagnosis, particularly when the “Mercedes Benz sign” is present, as it may suggest a rare cholecysto-hydatid cyst fistula. However, this is not always the case. Prompt and accurate evaluation, including intraoperative findings, to reinforce clinical suspicion and decision-making in endemic regions.

## Highlights

• The presence of gas in the gallstones, known as the Mercedes-Benz sign, led to the initial misdiagnosis of a cholecysto-hydatid cyst fistula. This rare diagnostic sign raised suspicion of a fistula between the liver hydatid cyst and the gallbladder, which was later found to be incorrect during surgery.
• Emergency surgery revealed acute cholecystitis and two hydatid cysts in liver segments IVb and VII, without the suspected cholecysto-hydatid fistula. The surgical procedures were crucial for the patient's recovery and successful outcome.
• Preoperative evaluation and accurate diagnosis were mandatory to avoid unnecessary surgical procedures. It also highlights the importance of a high index of suspicion for complex conditions like hydatid cyst complications, especially in endemic regions.

## 1 Introduction

Liver hydatid cyst is considered a cosmopolitan disease due to its widespread occurrence across the globe [1]. While it may be more prevalent in endemic regions, it is not limited to specific geographical areas, making it a global health concern. In endemic countries, the frequency of liver hydatid cyst cases is notably high, contributing to its status as a significant public health issue. Despite being categorized as a relatively mild disease in its early stages, liver hydatid cysts can lead to severe complications, some of which are life-threatening. One such complication is the formation of a fistula, a connection between the cyst and the bile ducts [2]. However, certain radiologic signs, such as the “Mercedes Benz sign,” indicating the presence of gas in gallstones, can lead to diagnostic errors. Here, we describe a case, according to SCARE guidelines [3], in which a wrong diagnosis of a rare instance of a cholecysto-hydatid cyst fistula is indicated by the presence of gas in the gallstones.

## 2 Case presentation

**Fig. 1:**
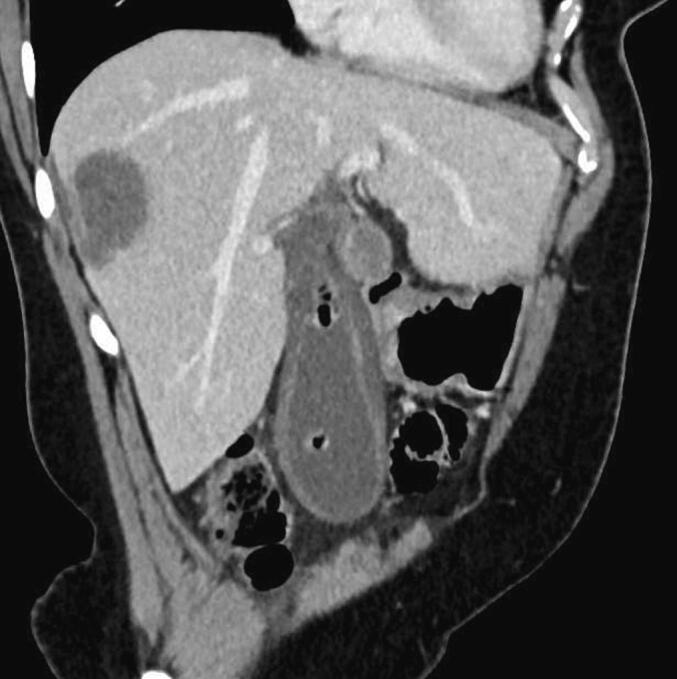
CT images showing the distended gallbladder with a thick wall and a gas in the gallstones.

**Fig. 2:**
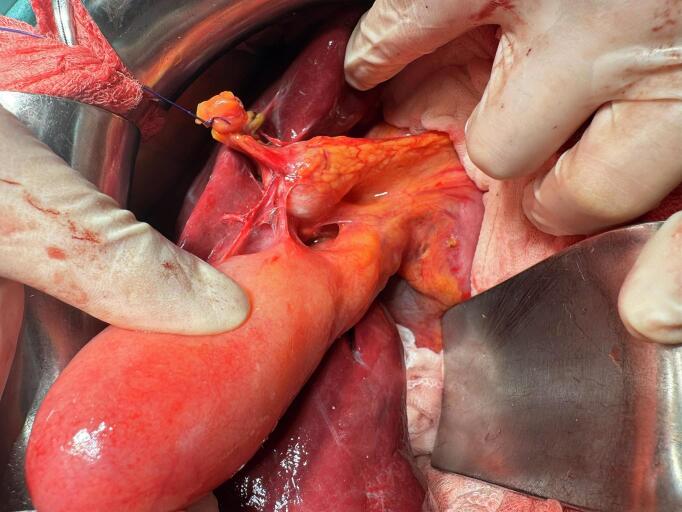
Intra-operative view of the gallbladder and the hydatid cyst of segment IVb.

**Fig. 3:**
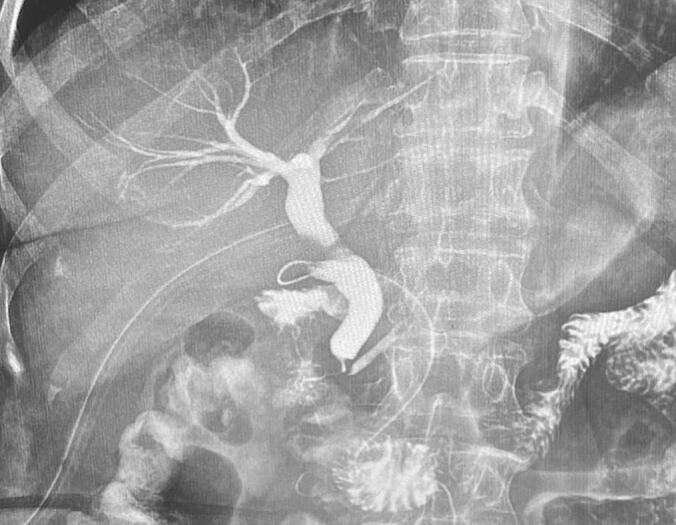
Intraoperative cholangiography after suturing the fistula.

**Fig. 4:**
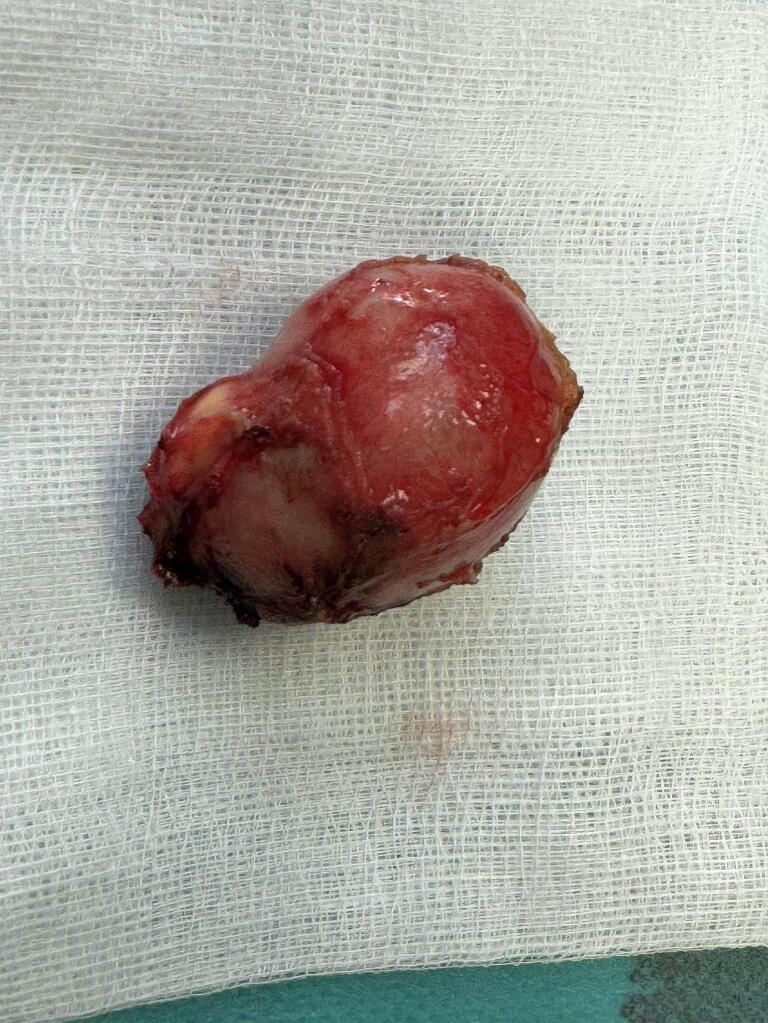
The operative specimen of the hydatid cysts after the perikystectomy.

A 30-year-old female patient with no preexisting conditions presented with right hypochondrium pain accompanied by a fever for 24 h. The patient is from a rural region and had contact with animals, especially dogs. On physical examination, the patient was febrile (39 °C), with guarding in the right upper quadrant of the abdomen. A palpable gallbladder was noted. The patient underwent an abdominal ultrasonography which revealed acute cholecystitis with the incidental discovery of two multilocular cystic formations in segments IVb and VII suggesting hydatid cysts. An abdominopelvic CT scan revealed intravesicular gas with close contact with the hydatid cyst of segment IVb, suggesting a cholecysto-hydatid fistula (Fig. 1). The gallbladder was distended 10*5 cm with a thickened wall of 8 mm. The patient underwent emergency surgery via a subcostal approach. Intraoperatively, a distended gallbladder with thick walls and two hydatid cysts were found, one in segment IV (Fig. 2) and the other in segment VII. There was no cholecysto-hydatid cyst fistula. The first surgical step was cholecystectomy and cholangiography through a transcystic drain, which did not show a cholecystobiliary fistula (Fig. 3). The second step involved total pericystectomy of the cyst in segment IVb (Fig. 4) and resection of the protruding dome of the cyst in segment VII after freeing the right liver. The resection of the first liver hydatid cyst revealed a cysto-biliary communication. This communication was sutured to avoid biliary leakage. Postoperative recovery was uneventful. The patient was discharged after two days under Albendazol (400 mg per day) for two weeks. After three months of follow-up, there was no recurrence of the hydatid disease.

## 3 Discussion

Liver hydatid cyst is a cosmopolitan disease [4]. It is still endemic in Tunisia and represents a health problem in developing countries [5]. The liver (70–80 %) and lungs (15–25 %) are the most common sites of hydatid cysts, but the involvement of other organs is also possible [6]. Hepatic hydatid cysts are usually asymptomatic. However, they may rupture, causing anaphylactic shock or fistulation. Rupture to the biliary tree is the most common complication, seen in 5 % to 15 % of cases. It is seen in the right liver in 55 % to 60 % of cases, in the left liver at 25 % to 30 %, and rarely in the junction point or gallbladder [7]. The cause for this appears to be the inclusion of biliary radicals into the pericyst during growth [8]. The presence of gas within gallstones has been known for over 200 years. The meaning of this finding is still debated [9] and it can support various diagnoses and signs. The presence of gas in the gallbladder, indicated by the “Mercedes Benz sign,” can be misleading and is often associated with a cholecysto-hydatid fistula, which was suspected in our case. The Mercedes Benz sign, and its presence in our case, raises suspicion of a fistula between the hydatid cyst of the liver and the gallbladder. It also known as the seagull sign refers to the typical triradiate pattern seen on imaging performed for diagnosing gallstones [1,10,11]. Despite the rarity of this sign, it serves as an important diagnostic clue, though its specificity remains questionable, leading to potential diagnostic challenges. It is a rare sign visible on X-ray and computerised tomography (CT) of the abdomen when sections are studied along the longitudinal axis of the calculus [12]. The gas in the fissures typically comprises less than 1 % oxygen, 6 %–8 % carbon dioxide, and the rest nitrogen [12]. The high concentrations of carbon dioxide and low oxygen relative to those in normal air may be due to the diffusion of gas into the calculus that may originate from a gas-forming organism [12] that was an infected hydatid cyst. In our case, the initial suspicion of a cholecysto-hydatid fistula was ultimately disproven during surgery, highlighting the need for careful intraoperative evaluation. While the rupture of hydatid cysts into the biliary tree is the most common complication, the involvement of the gallbladder is rare and adds complexity to the clinical presentation. This case underscores the importance of differentiating between the radiological signs of hydatid cyst complications and other causes of gas formation in the biliary system to avoid misdiagnosis.

## 4 Conclusion

In conclusion, the appearance of a fistula between the hydatid cyst of the liver and the gallbladder represents a complex and challenging medical condition. However, certain radiologic signs, such as the “Mercedes Benz sign” indicating the presence of gas in the gallbladder, can mislead the diagnosis and complicate the decision. Clinicians should maintain a high index of suspicion for this condition, particularly in regions where liver hydatid cysts are endemic for better decision-making.

## Patient consent

Written informed consent was obtained from the patient to publish this case report and accompanying images. On request, a copy of the written consent form is available for review by the editor-in-chief of this journal.

## Provenance and peer review

Not commissioned, externally peer-reviewed.

## Ethical approval

Ethical approval is exempt/waived at our institution for all the case reports.

## Funding

No funding.

## Guarantor

Mohamed Ali Chaouch.

## Research registration number

Not applicable.

## CRediT authorship contribution statement

All the authors participated in the manuscript and validated the final version of the manuscript

## Declaration of competing interest

The authors declare no competing interest.
